# Acute heart failure due to coronary subclavian steal syndrome after coronary artery bypass grafting

**DOI:** 10.1002/ccr3.7326

**Published:** 2023-05-09

**Authors:** Kosuke Nakata, Kosaku Nishigawa, Takashi Yoshinaga, Toshihiro Fukui

**Affiliations:** ^1^ Department of Cardiovascular Surgery Kumamoto University Hospital Kumamoto Japan

**Keywords:** acute heart failure, axillo‐axillary bypass grafting, coronary artery bypass grafting, coronary subclavian steal syndrome, subclavian artery occlusion

## Abstract

**Key clinical message:**

An axillo‐axillary bypass grafting is useful for coronary subclavian steal syndrome when occlusion of the proximal left subclavian artery.

**Abstract:**

An 81‐year‐old female who had undergone coronary artery bypass grafting 15 years previously was admitted and diagnosed with coronary subclavian steal syndrome. Preoperative angiography showed backflow from the left anterior descending coronary artery to the left internal thoracic artery and occlusion of the proximal left subclavian artery. Axillo‐axillary bypass grafting was successfully performed.

## 1 INTRODUCTION

Coronary subclavian steal syndrome (CSSS) is an uncommon complication of coronary artery bypass grafting (CABG) using the internal thoracic artery (ITA). This could be explained by the fact that a proximal subclavian artery (SCA) stenosis or occlusion causes retrograde blood flow from the native coronary artery to the ITA. CSSS can be associated with angina, acute coronary syndrome, and sudden cardiac death, despite the patency of the grafted vessels. We experienced an 81‐year‐old female patient with acute heart failure due to CSSS, which was successfully treated with axillo‐axillary bypass grafting (AABG) surgery.

## 2 CASE

**FIGURE 1:**
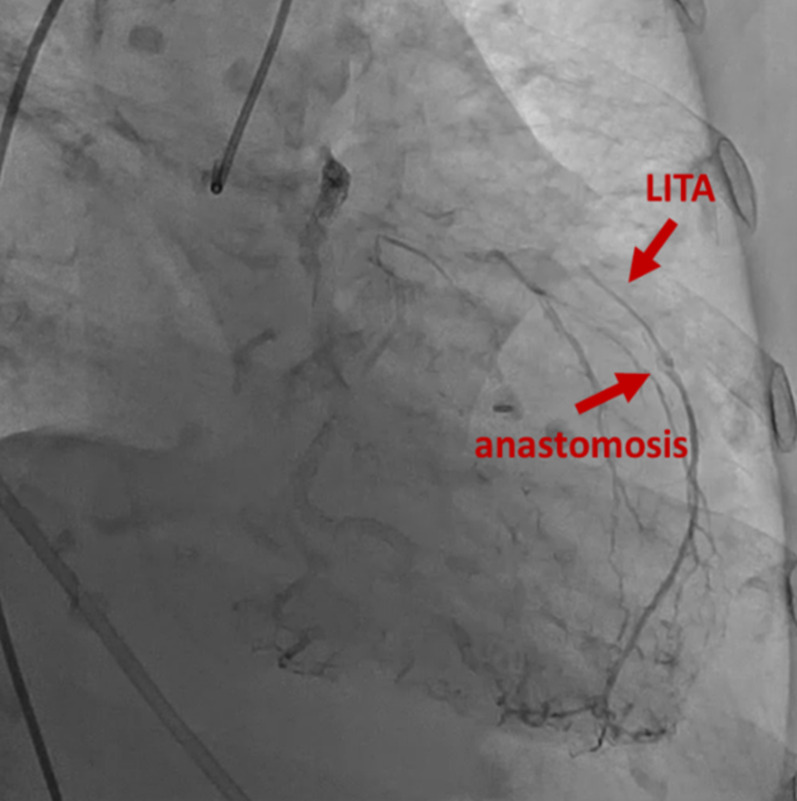
Coronary angiography showed that the left anterior descending coronary artery (LAD) was to & fro, and backflow from the LAD to the left internal thoracic artery.

**FIGURE 2:**
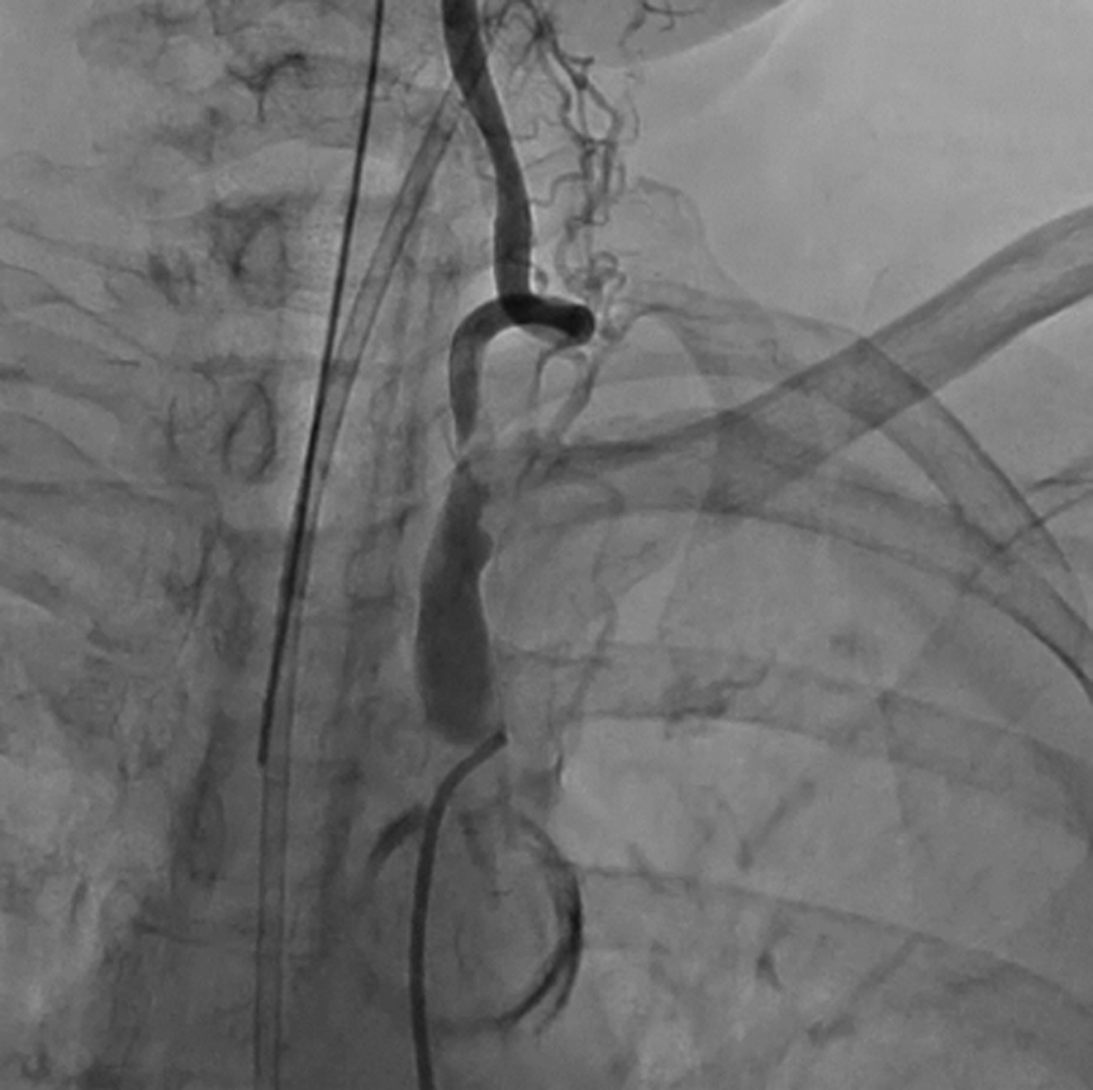
The left subclavian artery was occluded near the bifurcation of the left vertebral artery.

An 81‐year‐old female had undergone CABG 15 years ago, which included the left ITA (LITA) to the left anterior descending coronary artery (LAD) and saphenous vein graft (SVG) to the left circumflex artery (LCX). After the operation, she received multiple percutaneous coronary interventions in the right coronary artery (RCA) and LCX because the SVG was occluded. She was transported to previous hospital due to chest discomfort and hypoxia at rest. Physical examination revealed the difference in right and left brachial systolic blood pressure (right; 179 mmHg vs. left; 104 mmHg), lung course crackles, tibial edema, and pleural effusions. Her saturation of percutaneous oxygen was 67%. She required mechanical ventilation support with tracheal intubation due to acute respiratory failure. Myocardial infarction was suspected and she was transported to referring hospital. Laboratory data revealed elevated brain natriuretic peptide (BNP) (BNP of 3400 pg/mL and NT‐proBNP of 16,172 pg/mL). Echocardiography showed decreased ejection fraction (EF) of 48.5%, and moderate mitral regurgitation (MR). An angiographic study showed the patent RCA and LCX with collateral circulations from them to the distal LAD, which competed with the blood flow from the LITA (Figure 1). Although the LITA was patent, the proximal left SCA (LSCA) was occluded near the bifurcation of the left vertebral artery (Figure 2). With these findings, she was diagnosed with CSSS with decreased blood flow from the LITA and referred to our department for surgical treatment. Heart failure was treated with diuretics. Hypoxia was improved and she was extubated.

**FIGURE 3:**
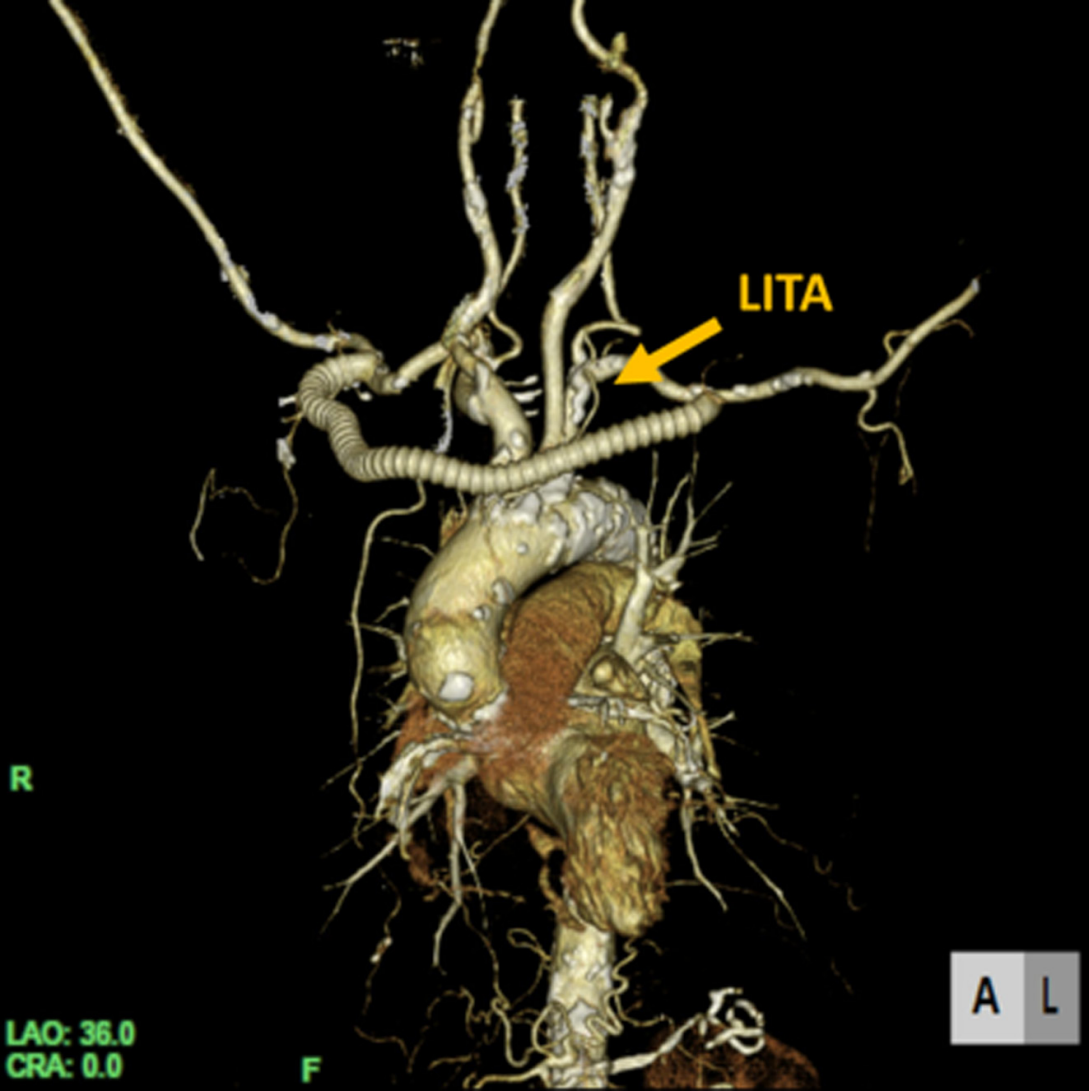
Postoperative contrast‐enhanced computed tomography revealed that the artificial blood vessel patented and anastomosis site had no abnormalities.

It was considered difficult to perform an endovascular treatment (EVT) to repair the LSCA because of severe calcification in the LSCA detected by contrast‐enhanced computed tomography. Magnetic resonance imaging (MRI) showed 90% stenosis in the left internal carotid artery and 50% stenosis in the right internal carotid artery. Then, AABG using an 8‐mm ringed polytetrafluoroethylene vascular prosthesis (PROPATEN: W.L. Gore & Associates) was successfully performed. The postoperative course was uneventful. Postoperatively, her symptoms disappeared and she was able to proceed without complications. The difference in systolic blood pressure disappeared (right; 126 mmHg vs. left; 127 mmHg). Postoperative laboratory data showed decrease of BNP (155 pg/mL) and NT‐proBNP (2600 pg/mL). Postoperative echocardiography showed that EF improved to 55.1% and MR improved to trivial. Contrast‐enhanced computed tomography confirmed the patency of the graft (Figure 3). Chest discomfort disappeared at postoperative month 3.

## 3 DISCUSSION

CSSS is a rare complication affecting approximately 0.2%–6.8% of patients who have undergone CABG. Stenosis or occlusion of the proximal part of the SCA reduces blood flow in the ITA and may cause CSSS. CSSS presents with symptoms of angina pectoris, acute coronary syndrome, and intubation caused by heart failure exacerbation, as in our case.^1^

CSSS is diagnosed when retrograde flow is observed in the grafted coronary artery and ITA in angiography.^1^ The grade of subclavian steal syndrome is classified according to the degree of stenosis of SCA as follows; (a) decreased blood flow velocity during mid‐systole, (b) to‐and‐fro finding retrograde flow during systole, (c) complete retrograde flow. Even though when there is to‐and‐fro flow during systole at rest, it can sometimes become a complete retrograde flow during angina.^1^, ^2^

Recently, EVT such as percutaneous angioplasty and endovascular stent placement has become the main treatment of choice, and surgical treatment is chosen only for selected patients.^3^ However, some studies demonstrated that surgical treatment was superior to EVT. Galyfos et al.^4^ reported no difference in 30 days postoperative patency between EVT and surgical treatment. On the other hand, long‐term patency rates, including 95% versus 89% at 1 year, 91% versus 83% at 3 years, and 87% versus 75% at 5 years, were significantly superior after surgical treatment.^4^ In fact, the European Society of Cardiology published in 2017 changed a case by case and both surgical revascularization and EVT options should be considered.^5^

There are several options for surgical revascularization: AABG, carotid‐subclavian artery bypass, and carotid‐carotid artery bypass.^1^, ^4^ In our case, angiography showed severe stenosis of the LSCA extending to the origin of the left vertebral artery, and MRI revealed stenosis of both internal carotid arteries. Therefore, the right axillary artery was selected for the inflow of the bypass graft. The distal anastomosis was placed in the LSCA distal to the carotid thyroid artery, which was thought to be the supply vessel, because there was a risk of myocardial ischemia due to decreased flow of the LITA caused by clamping the axillary artery during anastomosis. Therefore, it is apparent that AABG is useful for CSSS.

## 4 CONCLUSIONS

We experienced a case of CSSS with acute heart failure due to LSCA occlusion. AABG surgery for CSSS provided good results. SCA stenosis or occlusion should be considered as a cause of myocardial ischemia after CABG.

## AUTHOR CONTRIBUTIONS

**Kosuke Nakata:** Conceptualization; writing – original draft. **Kosaku Nishigawa:** Writing – review and editing. **Takashi Yoshinaga:** Writing – review and editing. **Toshihiro Fukui:** Supervision.

## FUNDING INFORMATION

This research was not externally funded.

## CONFLICT OF INTEREST STATEMENT

The authors declare no conflicts of interest.

## CONSENT

Written informed consent was obtained from the patient to publish this report in accordance with the journal's patient consent policy.

## Data Availability

The data that support the findings of this study are openly available in pubmed.gov at https://pubmed.ncbi.nlm.nih.gov/, reference numbers 1 to 5.
